# 19751 Identifying Barriers to Diabetes Technology in Low-Income, Type 1 Patients

**DOI:** 10.1017/cts.2021.763

**Published:** 2021-03-30

**Authors:** Emily Donahue, Apurva Uniyal, Terry David Church

**Affiliations:** 1 University of Southern California; 2 USC School of Pharmacy

## Abstract

ABSTRACT IMPACT: This research will aid clinical and policy solutions on lessening the vast health disparities and overall access issues for low-income, type 1 diabetes patients. OBJECTIVES/GOALS: Identify key barriers to accessing continuous glucose monitors (CGMS) and care options for low-socioeconomic status (SES) patients on public insurance. Low-SES patients with type 1 diabetes (T1D) have lower utilization rates of effective diabetes management technologies and worse clinical outcomes. METHODS/STUDY POPULATION: A literature review was conducted to understand the current research landscape for T1D and lead to the identification of potential barriers which included socioeconomic status, low-income, health literacy, and racial/ethnic minority. Clinicaltrials.gov was searched using the keyword ‘type 1 diabetes’ in conjunction with the identified barriers (as well as the keyword ‘barrier’). A follow up review of each state’s Medicaid programs was conducted to analyze cost and access options for CGMs and the overall financial burden of the disease on low-SES T1D patients. States that offered CGM coverage were further analyzed to determine reimbursement rates and actual out-of-pocket cost for patients. RESULTS/ANTICIPATED RESULTS: Of 285 trials identified from Clinicaltrial.gov searches, only seven relevant trials examined barriers and T1D for low-SES patients. Additionally, many of these studies, both in and outside of the clinical trial space, seldom distinguished between type 1 and type 2 diabetes’‘ an important distinction given that T1D has a higher financial burden and a quicker onset of complications. Currently, 39 states offer various insurance coverage through their Medicaid programs, but have clinical restrictions and requirements such as pediatric coverage only or minimum blood glucose requirement checks. Additionally, there is vast variability in reimbursement rates between states ($0-$800). DISCUSSION/SIGNIFICANCE OF FINDINGS: Study results indicate less effective diabetes management for low-SES T1D patients and a need for more intersectional clinical trial research. Differences in state’s Medicaid CGM coverage, expressed in disparate clinical outcomes for these T1D patients, belies financial incentives to health improvements, as annual US T1D costs are $14.4 billion.

